# PM2.5 Induced Nasal Mucosal Barrier Dysfunction and Epithelial–Mesenchymal Transition to Promote Chronic Rhinosinusitis Through IL4I1-AhR Signaling Pathway

**DOI:** 10.3390/toxics13060488

**Published:** 2025-06-10

**Authors:** Yue Wang, Bowen Zheng, Panhui Xiong, Yijun Liu, Longlan Shu, Yang Shen, Tao Lu, Yucheng Yang

**Affiliations:** 1 Department of Otorhinolaryngology, Upper Airway Inflammation and Tumor Laboratory, The First Affiliated Hospital of Chongqing Medical University, Chongqing 400016, China; wangyuexbb@163.com (Y.W.); 1474406929@proton.me (B.Z.); xph@stu.cqmu.edu.cn (P.X.); liuyijun@scu.edu.cn (Y.L.); sllent2021@163.com (L.S.); 203421@hospital.cqmu.edu.cn (Y.S.); 2 Department of Otorhinolaryngology-Head and Neck Surgery, The First Affiliated Hospital of Ningbo University, Ningbo 315000, China

**Keywords:** PM2.5, aryl hydrocarbon receptor, Interleukin 4 Induced 1, nasal mucosal barrier, epithelial–mesenchymal transition, chronic rhinosinusitis

## Abstract

Environmental pollutants like PM2.5 contribute to chronic rhinosinusitis (CRS). The aryl hydrocarbon receptor (AhR), a contaminant sensor linked to tryptophan metabolites, is regulated by IL4I. However, how PM2.5 stimulation via IL4I1 influences AhR activation and CRS pathogenesis remains unclear. This study explored the IL4I1-AhR pathway in CRS using patient tissues, HNEpCs, and murine models. Methods included IHC, qRT-PCR, and WB under PM2.5 exposure, with further investigation into downstream effects on CYP1B1 and epithelial–mesenchymal transition (EMT). Significant upregulation of IL4I1, AhR, and CYP1B1 was observed in CRS tissues, with higher expression levels in CRS patients. Exposure to PM2.5 activated the IL4I1-AhR pathway, leading to decreased E-cadherin, increased N-cadherin and vimentin, and impaired nasal mucosal barrier function. In vitro experiments demonstrated that PM2.5-induced EMT in HNEpCs was mediated by IL4I1-dependent AhR activation. CH223191 reduced cell migration and EMT, while IL4I1 knockdown attenuated AhR activation and EMT marker expression. Murine models further confirmed that PM2.5 exacerbated nasal polyp formation and tissue remodeling via the IL4I1-AhR pathway. This study underscores the critical role of the IL4I1-AhR signaling pathway in PM2.5-induced nasal mucosal barrier dysfunction and EMT in CRS. IL4I1, as an upstream regulator of AhR, promotes EMT and nasal mucosal barrier disruption.

## 1. Introduction

Chronic rhinosinusitis (CRS) is a prevalent, approximately 12–15%, chronic inflammatory disease of the sinus mucosa, classified into chronic rhinosinusitis with nasal polyps (CRSwNP) and chronic rhinosinusitis without nasal polyps (CRSsNP) phenotypes [1]. The pathogenesis of CRS remains poorly understood, but the immune barrier hypothesis proposes that nasal epithelium damage plays a key role. When irritated, nasal cells show thickened membranes, excess mucus cell growth, and abnormal blood vessel formation—all weakening nasal defenses. [2,3]. These disrupts the epithelial barrier, causing immune dysfunction and chronic inflammation, as outlined in the 2020 European Position Paper on Rhinosinusitis and Nasal Polyps (EPOS) [1,2]. The pathogenesis of CRS is multifactorial, involving environment, climate, and lifestyle. Pollutants, particularly PM2.5, increased CRS prevalence and severity by damaging the nasal barrier, triggering immune responses, and causing tissue remodeling [4,5,6].

The aryl hydrocarbon receptor (AhR), a ligand-activated transcription factor that plays a crucial role in xenobiotic metabolism and immune regulation [7]. In its inactive state, AhR resides in the cytoplasm as part of a multiprotein complex [8,9,10], but upon binding to ligands like PM2.5 or tryptophan (Trp) metabolites, it translocates to the nucleus to induce the expression of detoxifying enzymes (CYP1A1 and CYP1B1) [11,12,13] and modulates various physiological processes, including inflammation, oxidative stress, and epithelial–mesenchymal transition (EMT) [14]. AhR ligands are classified into exogenous and endogenous categories (e.g., Polycyclic Aromatic Hydrocarbons (PAHs) in PM2.5) [15], and endogenous (e.g., Trp metabolites) categories. Studies have shown the enrichment of Trp metabolism pathways within the CRS sinonasal microbiota [16]. Key enzymes involved in Trp catabolism include IDO1, IDO2, TDO2, TPH1, TPH2, IL4I1, and DDC. In CRS, IDO1, TDO2, and KYN influence reactive oxygen species (ROS) through AhR activation [17]. Interleukin 4 Induced 1 (IL4I1), a metabolic enzyme, converts Trp to AhR-activating metabolites (indole-3-pyruvate, and indole-3-aldehyde) [18]. Recent studies have indicated that pollutants alter IL4I1 expression, impacting sperm motility [19], while macrophage-derived IL4I1 worsens allergic rhinitis by promoting nasal EMT [20]. Although the role of IDO1-catalyzed KYN in CRS is known, the involvement of IL4I1 remains unclear.

In environmentally mediated inflammatory and respiratory diseases, AhR activation by PAHs disrupts mucosal barriers through oxidative stress and inflammation, leading to tissue damage in chronic obstructive pulmonary disease and asthma [11,21]. AhR regulates Th17/Treg cell balance and antigen-presenting cell responses, influencing respiratory inflammation. Th17-specific AhR activation promotes IL-22 production, exacerbating allergic airway inflammation [22,23]. AhR deficiency exacerbates pulmonary inflammation via NF-κB-mediated barrier disruption [24]. Pollutants disrupts epithelial cell junctions, leading to EMT, characterized by lost epithelial traits and gained mesenchymal features, which compromises mucosal integrity [25,26,27]. PM2.5 activates AhR in CRS, triggering EMT and disrupting nasal epithelial barriers, leading to tissue remodeling [28]. Benzo[a]pyrene (BAP) activates AhR in the airways, promoting ROS production, upregulating mucin expression, and exacerbating airway inflammation and barrier dysfunction in asthma [25]. Similarly, cigarette smoke upregulates CYP1A1, CYP1B1, and MMP-1, degrading collagen and remodeling barriers [29]. AhR activation by LPS/IFN-γ in macrophages reduces IL-6 and IL-12, impairing barrier function [30], while EMT is implicated in CRS [31,32]. How PM2.5 drives CRS through IL4I1 and AhR remains unclear. This study aims to investigate how PM2.5 stimulates human nasal mucosal epithelial cells, activates IL4I1, modulates AhR expression, and induces nasal mucosal damage and tissue remodeling, thereby contributing to nasal polyp formation.

## 2. Materials and Methods

### 2.1. Patient Selection Criteria

Patients were recruited from the Department of Otorhinolaryngology. The study enrollment period spanned from 1 January 2023 to 31 December 2023. Participants were selected based on EPOS2020 diagnostic criteria [1], with all cases pathologically confirmed before surgery. The inclusion criteria comprised the following:

(1) Patients with no prior history of sinus surgery.
(2) Patients without a history of lower respiratory tract diseases, including but not limited to asthma and chronic bronchitis.
(3) Patients who had not received any relevant medications, such as nonsteroidal anti-inflammatory drugs (NSAIDs) or anticoagulants, within a specified timeframe prior to the study.

Participants were categorized into three groups: CRSsNP, *n* = 20, CRSwNP, *n* = 20, and control *n* = 20. The control group comprised patients undergoing septoplasty without evidence of CRS. The research protocol was approved by the Ethics Committee, and written informed consent was obtained from all participants prior to their inclusion in the study.

### 2.2. Tissue Distribution and Preparation

Following surgical excision, tissue samples were rinsed three times with PBS and divided into three sections. One section was immediately utilized for the primary cell culture in vitro. The second section was embedded in paraffin and sectioned to a thickness of 4–5 μm for immunohistochemistry (IHC) analysis. The final section was stored at −80 °C for subsequent Western blot (WB) and qualitative real-time polymerase chain reaction (qRT-PCR) experiments.

### 2.3. Murine Model of CRS

**Figure 1:**
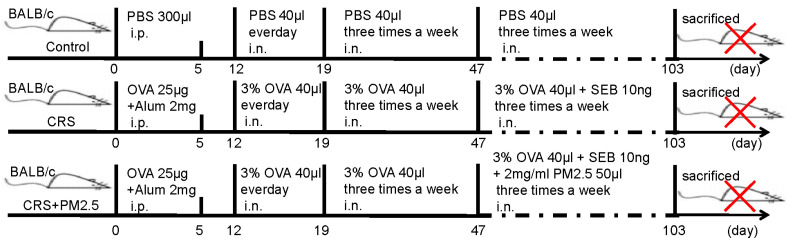
Graphical flowchart illustrating CRS and CRS combined with PM2.5 exposure. The red cross indicates that the mice were euthanized after the drug administration process.

To ensure experimental validity and reproducibility, stringent environmental controls were implemented throughout the study. All mice were kept in a standard SPF animal laboratory (Chongqing, China). The control group was maintained under normal, clean air conditions, while the experimental groups were exposed to varying concentrations of PM2.5. To develop a PM2.5-induced CRS mouse model, 4-week-old female BALB/c mice were sensitized with intraperitoneal Ovalbumin (OVA)/alum on days 0 and 5, followed by nasal OVA challenges from days 12–103. From day 47, selected mice received additional intranasal SEB plus PM2.5 exposure (3× weekly). This protocol combined allergic sensitization (OVA), bacterial toxin-induced inflammation (Staphylococcal Enterotoxin B (SEB)), and pollutant exposure (PM2.5) to mimic human CRS pathogenesis. Tissues were collected 24 h post-final exposure for analysis (Figure 1).

### 2.4. Immunohistochemistry

Tissue sections were immunostained with anti-AhR antibody (1:250, Abcam, Cambridge, UK), anti-IL4I1 antibody (1:100, Abcam, Cambridge, UK), anti-CYP1B1 antibody (1:1000, Proteintech, Wuhan, China), anti-E-cadherin antibody (1:1000, Proteintech, Wuhan, China), anti-N-cadherin antibody (1:10,000, Proteintech, Wuhan, China), and anti-vimentin antibody (1:5000, Proteintech, Wuhan, China). Antigen retrieval was achieved by treating the sections with citrate buffer. The slides were then incubated with a horseradish peroxidase-labeled polymeric anti-rabbit secondary antibody and counterstained with hematoxylin. Negative and positive controls were included in each experiment. Five randomly selected fields (400× magnification) per sample were examined under an optical microscope (Leica DM500 ICC50, Leica, Wetzlar, Germany).

### 2.5. Immunofluorescence Staining

For immunofluorescence staining, tissue sections were first blocked with PBT-1 followed by overnight incubation at 4 °C with primary antibodies (anti-AhR, 1:250, Abcam, Cambridge, UK). Subsequently, sections were treated with Alexa Fluor 647-conjugated secondary antibodies (1:1000; Invitrogen, Carlsbad, CA, USA) and DAPI (1:1000, Sigma-Aldrich, St. Louis, MO, USA) at room temperature for 1 h. After mounting, immunofluorescence signals were visualized using a laser scanning confocal microscope (Leica, Wetzlar, Germany).

### 2.6. Hematoxylin and Eosin (HE) Staining

HE staining was performed to confirm the pathological model of rhinosinusitis. Tissue sections, cut to 5 μm thickness, were stained with hematoxylin for approximately 4 min, rinsed with deionized water, and briefly immersed in hydrochloric acid alcohol. After a second rinse with deionized water, the sections were stained with eosin for 1 min. The sections were then dehydrated through a graded ethanol series, cleared in xylene, and mounted with resin mounting medium.

### 2.7. Masson Trichrome Staining

Masson trichrome staining was performed to differentiate collagen fibers, muscle, and other connective tissues. Sections were stained with Weigert’s iron hematoxylin for nuclear staining, followed by Biebrich scarlet-acid fuchsin for cytoplasm and muscle. Phosphotungstic and phosphomolybdic acids were used to treat the sections, and collagen was stained with aniline blue. After rinsing with acetic acid, the sections were examined under an optical microscope (Leica DM500 ICC50, Leica Camera AG, Wetzlar, Germany).

### 2.8. RNA Extraction and Real-Time Polymerase Chain Reaction

RNA was extracted with TRIzol (Invitrogen, Carlsbad, CA, USA) and reverse transcribed using RevertAid (Thermo Scientific, Waltham, MA, USA). qPCR used SYBR Green (TaKaRa, Kusatsu, Japan) with GAPDH control, performed in triplicate (95 °C/10 min; 40 cycles: 95 °C/15 s, 60 °C/15 s, 72 °C/30 s). Data were analyzed with RealPlex 2.5 software. Primers are shown in Supplementary Table S1.

### 2.9. Western Blotting

Tissue proteins were extracted using RIPA lysis buffer supplemented 1% PMSF. Protein concentrations were determined using a BCA protein assay kit (Beyotime, Shanghai, China). Proteins were separated by 10% SDS-PAGE and transferred onto PVDF membranes (Bio-Rad, Hercules, CA, USA). Membranes were blocked with 5% non-fat milk in TBST for 2 h at room temperature and then incubated with primary antibodies, including anti-AhR (1:250, Abcam, Cambridge, UK), and anti-IL4I1 (1:1000, Abcam, Cambridge, UK), CYP1B1 (1:1000, Proteintech, Wuhan, China), E-cadherin (1:5000, Proteintech, Wuhan, China), N-cadherin (1:10,000, Proteintech, Wuhan, China), and vimentin (1:20,000, Proteintech, Wuhan, China), at 4 °C overnight. Protein bands were visualized using BeyoECL Plus (Advansta, Menlo Park, CA, USA) after 1 h incubation with HRP-conjugated secondary antibodies (1:2000, Beyotime, Shanghai, China) at RT, and quantified using Quantity One v4.62 (Bio-Rad, Hercules, CA, USA).

### 2.10. Primary Culture of Human Nasal Mucosal Epithelial Cells (HNEpCs)

Nasal epithelial cells (HNEpCs) were isolated from CRS patients (both CRSwNP and CRSsNP subtypes) and control subjects during routine endoscopic sinus surgery under general anesthesia. Tissue specimens were transported to the laboratory on ice within 15 min of excision. After three washes with pre-cooled PBS, primary epithelial cells were isolated using enzymatic digestion followed by differential adhesion. The cell suspension was cultured in a 37 °C incubator with 5% CO_2_ until reaching approximately 90% confluence, at which point subsequent experiments were initiated.

### 2.11. Scratch Assay

Cells were seeded into 6-well plates (Corning, Corning, NY, USA) and allowed to adhere overnight. A scratch was created in the confluent cell monolayer using a 10 μL pipette tip (Biosharp, Hefei, China). After washing with PBS, a fresh culture medium containing 2% fetal bovine serum and experimental treatments was added. Cell migration into the scratch wound was monitored and photographed at regular intervals.

### 2.12. CCK-8 Assay

Cells in the logarithmic growth phase were harvested, rinsed with PBS, and dissociated into a single-cell suspension using trypsin. The cell concentration was determined using a cell counting chamber (Thermo Scientific, Waltham, MA, USA). Cells were plated in 96-well plates (Biosharp, Hefei, China) and cultured to adherence. After treatment, 10 μL of CCK-8 reagent was added to each well, and the plates were incubated for an additional hour. Optical density (OD) values were measured using a microplate reader (Thermo Scientific, Waltham, MA, USA) to assess cell viability and proliferation.

### 2.13. Protein–Protein Interaction (PPI) Network Construction

PPI data were obtained from STRING and analyzed using Cytoscape (v3.6.1) to construct the interaction network, with interactions represented as edges. Topological analysis was performed to identify key hub genes within the network.

### 2.14. Statistical Analysis

Statistical analysis was conducted using GraphPad Prism 8.3.0 software (GraphPad Software, San Diego, CA, USA), with data expressed as mean ± SD. For multi-group comparisons, we applied: (1) one-way ANOVA for normally distributed data with equal variances, (2) Welch’s ANOVA for normal distributions with unequal variances, and (3) Kruskal–Wallis test for non-normal distributions. A *p*-value < 0.05 was considered statistically significant.

## 3. Results

### 3.1. Activation of the AhR in CRS

**Figure 2:**
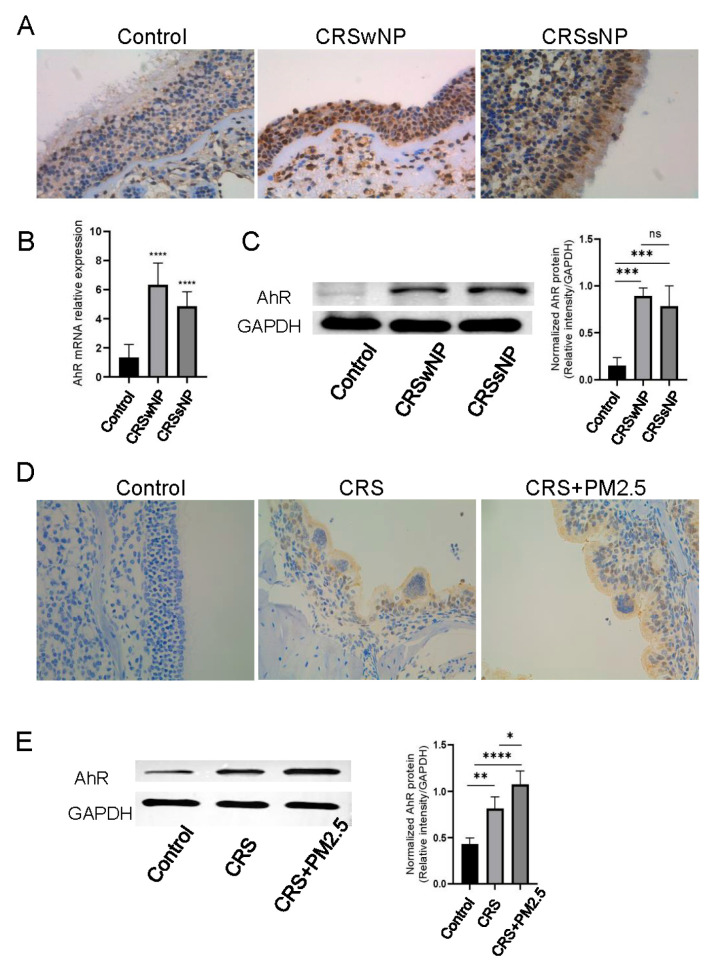
AhR Activation in CRS. (**A**) Immunohistochemistry staining of AhR in human nasal mucosal epithelium tissue sections revealed AhR expression, with significantly higher expression levels observed in CRS (including CRSwNP and CRSsNP) compared with control (×400 magnification). (**B**) mRNA expression of AhR in human nasal mucosal epithelium. The graph demonstrates elevated AhR expression in the CRS group (including CRSwNP and CRSsNP) relative to control. (**C**) Protein expression of AhR in human nasal mucosal epithelium. The bar chart indicates statistically significant upregulation of AhR in CRSwNP and CRSsNP compared with control, with CRSwNP showing higher expression than CRSsNP. (**D**) Immunohistochemistry staining of AhR in mouse nasal epithelium demonstrated strong AhR expression in both CRS and CRS + PM2.5 groups compared with control. (**E**) Western blot analysis confirmed significantly higher AhR expression in CRS and CRS + PM2.5 groups compared with control, with CRS + PM2.5 exhibiting greater expression than CRS, showing statistical significance. * *p* < 0.05, ** *p* < 0.01, *** *p* < 0.001, **** *p* < 0.0001; “ns” indicates no statistical significance.

To investigate the expression of AhR, nasal tissue samples were collected from patients. IHC staining of human nasal tissue sections revealed that AhR was expressed in control tissues, as well as in tissues from CRSwNP and CRSsNP. Notably, AhR expression was significantly stronger in CRSwNP and CRSsNP tissues compared with the control (Figure 2A). Consistent with these findings, both mRNA (Figure 2B) and protein (Figure 2C) expression levels of AhR were markedly upregulated in CRSwNP and CRSsNP tissues relative to the control. To further validate the effects of PM2.5 on AhR expression, a mouse model of CRS was established (Figure 1). IHC analysis of the model demonstrated that AhR was expressed in the nasal mucosal epithelium and was significantly elevated in both the CRS and CRS + PM2.5 groups compared with the control (Figure 2D). The protein expression level of AhR was significantly higher in both the CRS and CRS + PM2.5 groups compared with control. Moreover, AhR expression in the CRS + PM2.5 group was significantly higher than in the CRS group (Figure 2E).

### 3.2. The Elevated AhR Induces Morphological Alterations in the Nasal Mucosal Epithelium

**Figure 3:**
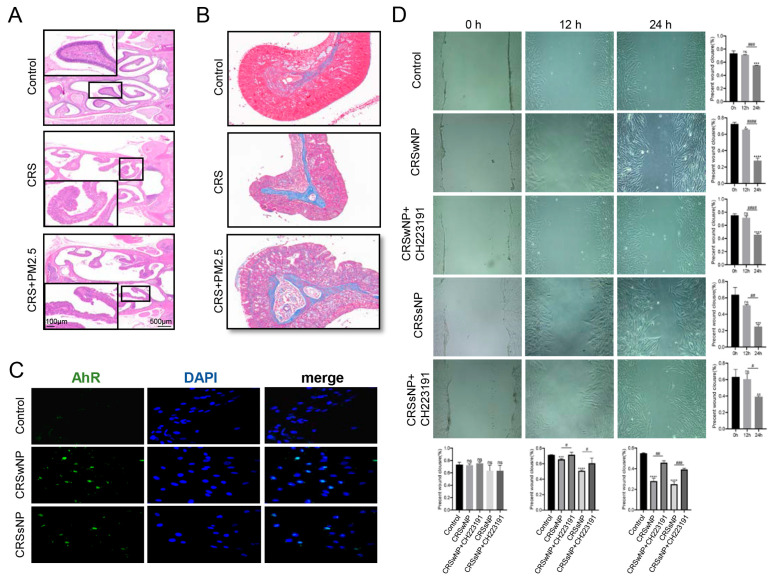
AhR Activation Triggers Cellular Morphological Remodeling. (**A**) Hematoxylin-eosin (H&E) staining of nasal cavity tissue sections revealed structural and morphological changes in the mouse nasal mucosal epithelium, primarily characterized by polypoid mucosal uplift in both the CRS and CRS + PM2.5 groups. The destruction of mucosal structure appeared more severe in the CRS + PM2.5 group compared with the CRS group. (**B**) Masson staining of mouse nasal mucosal tissue sections demonstrated abnormal proliferation and disorganized arrangement of collagen fibers. (**C**) Immunofluorescence staining indicated low expression of AhR in control cells and high expression in CRSwNP and CRSsNP cells, with AhR predominantly localized in the nucleus. AhR is shown in green, and cellular nuclei are stained blue with 4′,6-diamidino-2-phenylindole (DAPI) (×400 magnification). (**D**) Scratch assay of human nasal mucosal epithelial primary cells revealed faster cell growth in CRSwNP and CRSsNP compared with control, while growth was slower in cells co-cultured with the AhR inhibitor CH223191. Cells in the CRS group (including CRSwNP and CRSsNP) exhibited a long fusiform morphology. The bar chart demonstrated significantly enhanced wound healing capacity in CRSwNP and CRSsNP groups compared with control. However, co-culture with CH223191 markedly reduced wound healing ability in CRSwNP and CRSsNP cells. * *p* < 0.05, ** *p* < 0.01, *** *p* < 0.001, **** *p* < 0.0001 vs. control; # *p* < 0.05, ## *p* < 0.01, ### *p* < 0.001, #### *p* < 0.0001; “ns” indicates no statistical significance.

HE staining of the mouse model confirmed the presence of nasal polypoid changes in both the CRS and CRS + PM2.5 groups, with more severe pathological alterations observed in the CRS + PM2.5 group, under the upregulated expression of AhR (Figure 3A). Masson trichrome staining further demonstrated pathological destruction and abnormal collagen fiber proliferation in the nasal mucosal epithelium, consistent with tissue remodeling (Figure 3B). Moreover, IF staining of AhR was performed in HNEpCs to verify its expression and localization. The results revealed higher AhR expression in HNEpCs from both CRSwNP and CRSsNP groups, with AhR predominantly localized in the nucleus (Figure 3C), indicating its activated state in CRS. The growth and migration of HNEpCs were assessed using a scratch assay at 0, 12, and 24 h, with or without the AhR inhibitor CH223191. The results showed that HNEpCs from the CRSwNP and CRSsNP groups migrated faster than control, with cells adopting a long spindle-shaped morphology over time. Additionally, the migration speed of HNEpCs was significantly reduced in the presence of CH223191 (Figure 3D).

### 3.3. AhR Activation in Association with IL4I1

**Figure 4:**
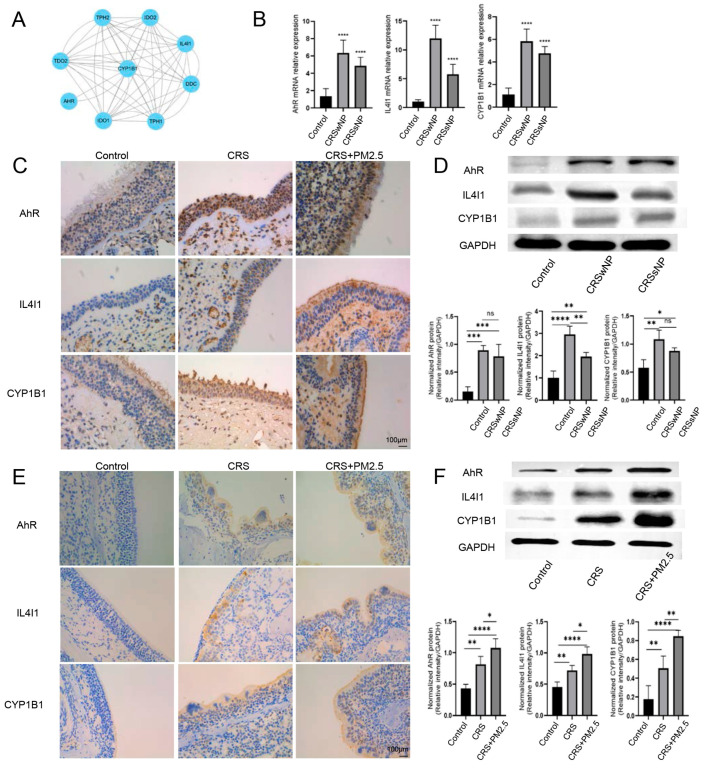
IL4I1-Mediated AhR Activation in CRS Pathogenesis. (**A**) A protein–protein interaction network. (**B**) mRNA expression of AhR, IL4I1, and CYP1B1 in human nasal mucosal epithelium. The graph depicts that AhR, IL4I1, and CYP1B1 all had higher expressions in the CRS group (including CRSwNP and CRSsNP). (**C**) Immunohistochemistry staining of AhR, IL4I1, and CYP1B1 in human nasal mucosal epithelium tissue sections. The figure shows the staining of AhR, IL4I1, and CYP1B1 expressed in human nasal mucosal epithelium, and the expressions of AhR, IL4I1, and CYP1B1 were all higher in CRS (including CRSwNP and CRSsNP) than those in control (×400 magnification). (**D**) Protein expression of AhR, IL4I1, and CYP1B1 in human nasal mucosal epithelium. The bar chart shows the statistically significant upregulation of AhR, IL4I1, and CYP1B1 in CRSwNP and CRSsNP compared with control, with IL4I1 expression higher in CRSwNP than in CRSsNP. (**E**) Immunohistochemistry staining of AhR, IL4I1 and CYP1B1 in mouse nasal epithelium. The results clearly depict that AhR, IL4I1, and CYP1B1 were strongly expressed in CRS and CRS + PM2.5 compared with control. (**F**) Western blot analysis of protein expression in mouse nasal epithelium. AhR, IL4I1, and CYP1B1 were significantly upregulated in CRS and CRS + PM2.5 groups compared with control, with higher expression in CRS + PM2.5 than in CRS. * *p* < 0.05, ** *p* < 0.01, *** *p* < 0.001, **** *p* < 0.0001, and “ns” is for no statistically significant.

To investigate the relationship between AhR and IL4I1, a PPI network was initially constructed utilizing data obtained from the STRING database (Figure 4A). The network featured CYP1B1 as a central seed node, along with eight interconnected nodes representing proteins that interact with CYP1B1. These interacting proteins included AhR, IDO1, IDO2, TDO2, TPH1, TPH2, IL4I1, and DDC. Network analysis revealed that TDO2, TPH1, TPH2, IDO1, and IDO2 exhibited expression patterns correlated with AhR through their interactions with CYP1B1. Additionally, IL4I1 was found to be associated with AhR expression via its connections to TDO2, TPH1, TPH2, IDO1, IDO2, and CYP1B1 (Supplementary Table S2).

Subsequently, to further explore the expression levels of IL4I1, AhR, and CYP1B1, qRT-PCR and WB analyses were conducted. The results demonstrated that the mRNA expression of IL4I1, AhR, and CYP1B1 was significantly elevated in both CRSwNP and CRSsNP compared with control (Figure 4B). IHC analysis of nasal mucosal tissue from CRS patients revealed the presence of IL4I1, AhR, and CYP1B1 in the human nasal epithelium (Figure 4C), with protein expression levels significantly higher in CRSwNP and CRSsNP compared with control (Figure 4D). Similarly, IL4I1, AhR, and CYP1B1 were detected in both the CRS and CRS + PM2.5 groups in mouse models (Figure 4E). Furthermore, protein levels of IL4I1, AhR, and CYP1B1 were consistently upregulated in CRSwNP and CRSsNP compared with control, and the protein expression of IL4I1, AhR, and CYP1B1 was significantly higher in the CRS + PM2.5 group compared with the CRS group alone.

### 3.4. PM2.5 Triggers the Activation of the IL4I1-AhR Signaling Pathway, Inducing EMT in CRS

**Figure 5:**
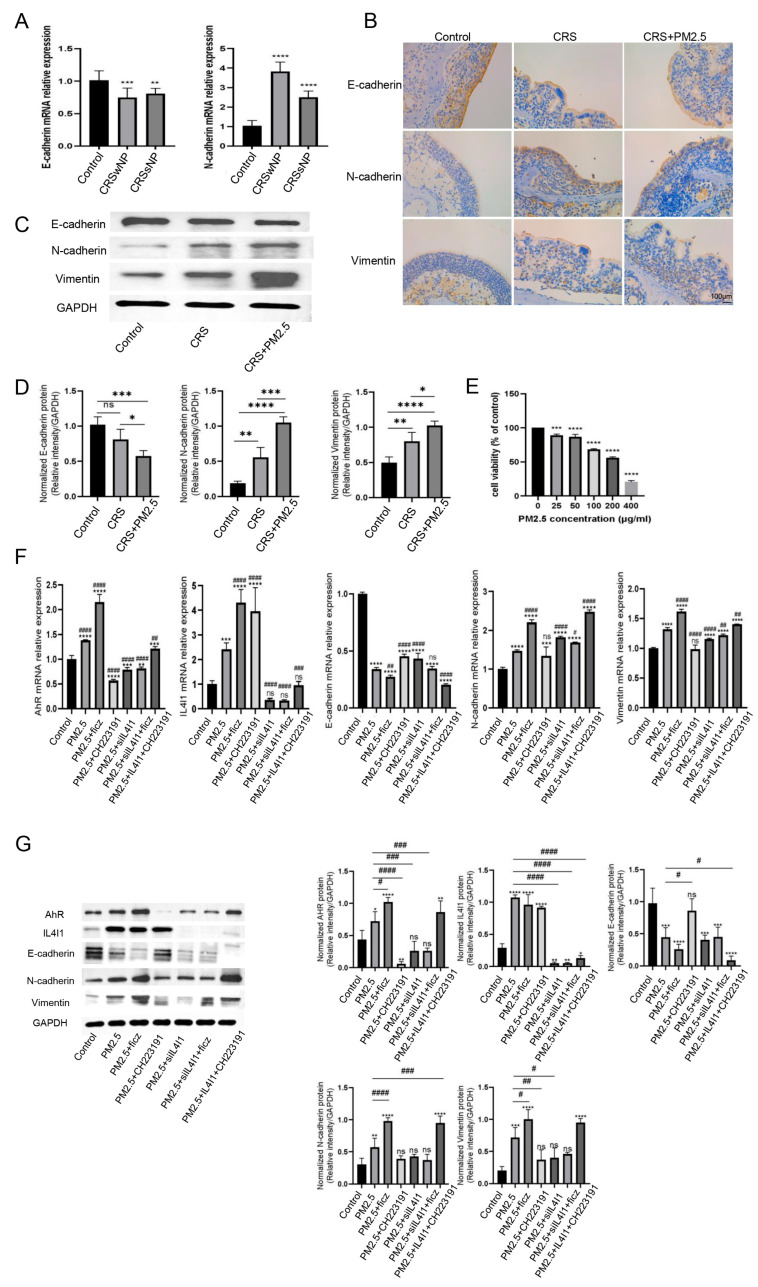
PM2.5-Induced IL4I1-AhR Signaling Drives Epithelial–Mesenchymal Transition in CRS. (**A**) mRNA expression of E-cadherin and N-cadherin in human nasal mucosal epithelium. E-cadherin was downregulated in CRSwNP and CRSsNP compared with control, while N-cadherin was upregulated. (**B**) Immunohistochemistry staining of epithelial–mesenchymal transition (EMT) markers (E-cadherin, N-cadherin, and vimentin) in mouse models. E-cadherin expression was weak in CRS and CRS + PM2.5 compared with control, while N-cadherin and vimentin were strongly expressed. (**C**,**D**) Western blot analysis of protein expression in mouse nasal epithelium. E-cadherin levels were significantly lower in CRS and CRS + PM2.5 compared with control, while N-cadherin and vimentin levels were higher. Differences between CRS and CRS + PM2.5 were statistically significant. (**E**) Determination of the optimal PM2.5 concentration for stimulating human nasal epithelial cells (200 μg/mL PM2.5 inhibited cell growth, reducing cell viability to approximately 50%). (**F**) qRT-PCR and (**G**) Western blot analysis of mRNA levels and protein expression of AhR, IL4I1, E-cadherin, N-cadherin, and vimentin. * *p* < 0.05, ** *p* < 0.01, *** *p* < 0.001, **** *p* < 0.0001, compared with the control; # *p* < 0.05, ## *p* < 0.01, ### *p* < 0.001, #### *p* < 0.0001, compared with PM2.5; and “ns” is for no statistically significant.

To validate the occurrence of EMT in our CRS patients, qRT-PCR was utilized. The bar charts illustrated that the mRNA expression of E-cadherin was significantly lower in CRSwNP and CRSsNP compared with the control, while N-cadherin exhibited an opposing trend (Figure 5A). Subsequently, the histological analysis of mouse nasal mucosa revealed disrupted epithelial integrity in CRS, characterized by altered expression of E-cadherin, N-cadherin, and vimentin. These alterations were more pronounced in the CRS + PM2.5 group. IHC staining further revealed reduced expression of E-cadherin and increased expression of N-cadherin and vimentin in both the CRS and CRS + PM2.5 groups, with more marked changes in the CRS + PM2.5 group (Figure 5B). WB analysis corroborated these findings, showing significant trends compared with control. Moreover, the changes observed in the CRS + PM2.5 group compared with CRS group were statistically significant (Figure 5C,D).

To further elucidate the role of PM2.5 in nasal mucosal epithelial barrier disruption, the optimal concentration of PM2.5 was determined using a CCK-8 assay. A concentration of 200 μg/mL PM2.5 was found to inhibit cell growth by approximately 50% (Figure 5E). qRT-PCR and WB revealed that PM2.5 exposure upregulated IL4I1 and AhR expression, accompanied by EMT markers indicative of a mesenchymal phenotype, including decreased E-cadherin and increased N-cadherin and vimentin (Figure 5F,G). Treatment with FICZ, an AhR agonist, elevated AhR but not IL4I1 levels, exacerbating EMT. Conversely, the AhR inhibitor CH223191 reduced AhR levels and mitigated EMT without affecting IL4I1. The knockdown of IL4I1 using siRNA-IL4I1 diminished both IL4I1 and AhR expression, attenuating EMT markers. Dual treatment with siRNA-IL4I1 and FICZ yielded results similar to siRNA-IL4I1 alone. Notably, CH223191 failed to inhibit AhR in the presence of IL4I1, suggesting that IL4I1 acts upstream of AhR in the signaling cascade. These findings collectively indicate that PM2.5 regulates EMT in nasal epithelial cells through the IL4I1-AhR signaling pathway.

## 4. Discussion

Our prior investigations elucidated that PM2.5 and di(2-ethylhexyl) phthalate (DEHP) activated the AhR within the nasal mucosa, disrupting the nasal mucosal epithelial barrier and contributing to CRS and allergic rhinitis [28,33,34]. DEHP acts through the AhR/cytochrome P450 enzyme pathway. In the current study, we demonstrate that PM2.5 upregulates IL4I1, which controls AhR/CYP1B1 signaling, leading to barrier dysfunction, EMT and CRS pathogenesis. Together, these findings indicated that AhR serves as a critical mediator of pollutant-induced nasal mucosal barrier dysfunction.

AhR signaling exhibits ligand-specific and cell-type-dependent responses, with Trp metabolites, inducing distinct conformational change that regulate diverse biological functions. Through bioinformatics, we identified the functional association of IL4I1 with AhR in the Trp metabolic network along with IDO1, IDO2, TDO2, TPH1, TPH2, and DDC. These enzymes modulate immune responses and inflammatory processes through tryptophan catabolism, generating bioactive metabolites that critically influence epithelial barrier dysfunction and tissue remodeling [35,36,37,38,39,40,41,42]. Recent studies have demonstrated that IL4I1 activates AhR more potently than IDO1 and TDO2 [18], generating indole metabolites and quinolinic acid that promote tumor progression in gliomas and leukemia by enhancing cancer cell motility and suppression immunity. While IDO1 and TDO2 initiate the KYN-AhR pathway, influencing mast cell activation in CRSwNP [17], especially given IDO1 inhibitors’ clinical failure. In CRS, we found upregulated IL4I1, AhR, and CYP1B1 in the nasal epithelial, with AhR nuclear translocation confirming pathway activation. IL4I1 promotes Trp catabolism and upregulates CYP1A1, accumulating metabolites in dendritic cells [43]. Furthermore, hCG induces IL4I1 in human endometrial epithelial cells, activating AhR through Trp metabolism to promote decidualization [44]. Thymol and anti-PD-1 antibodies therapy suppresses IL4I1 and AhR signaling in lung adenocarcinoma models [45]. Beyond activation by endogenous or exogenous factors, mechanical stress enhances hepatocyte IL4I1, activating AhR signaling to drive tumor progression and recurrence [46].

AhR serves as a key sensor and effector molecule for environmental pollutants such as PM2.5, playing a significant role in the development and progression of inflammatory and respiratory diseases through the mediation of oxidative stress, inflammatory responses, and immune regulation. This process leads to significant increases in intracellular ROS, mitochondrial ROS, and DNA damage [47]. PM2.5 impairs barrier integrity by inhibiting AhR-dependent tight junction protein, contributing to airway diseases [48,49]. Additionally, PM2.5 disrupts the balance of Th17/Treg cells via AhR, further contributing to pulmonary function injury and histopathological damage [50,51]. In our present study, PM2.5 was found to disrupt the integrity of the nasal mucosal epithelium in vitro and murine models, leading to tissue remodeling and exacerbating polypoid changes. This effect was accompanied by significant upregulation of AhR, IL4I1, and CYP1B1, as well as the induction of EMT. This is consistent with existing studies. A substantial body of research has established a strong association between EMT and the pathogenesis of CRS [52]. Our prior research has further corroborated these findings, revealing differential expressions of E-cadherin, vimentin, and α-SMA in CRS tissues and cells compared with control, across experimental systems [53]. These changes were corroborated in HNEpCs treated with PM2.5. These results collectively suggest that PM2.5 induces EMT in nasal mucosal epithelial cells by activating the IL4I1-AhR signaling pathway, ultimately leading to the disruption of the nasal mucosal barrier integrity. AhR activation induces the expression of cytochrome P450 enzymes, such as CYP1A1 and CYP1B1, leading to the excessive generation of ROS. The accumulation of ROS not only causes direct cellular damage but also activates inflammation-related signaling pathways, including NF-κB and MAPK, thereby further promoting EMT. Additionally, AhR activation promotes EMT by upregulating pro-inflammatory cytokines (IL-6, TNF-α) to create an inflammatory microenvironment. AhR also exhibits intricate interactions with the TGF-β signaling pathway. Recent studies also have highlighted that PM2.5 variously triggers AhR activation to promote EMT. PM2.5 contains various PAHs and other AhR ligands that directly activate the AhR signaling pathway, or upregulate pro-inflammatory cytokines (IL-6, TNF-α) to create an inflammatory microenvironment [54,55,56].

Emerging research has identified a novel immunometabolic axis in CRS pathogenesis involving Trp catabolism and AhR signaling. Clinical studies demonstrate detectable Trp levels in CRS patients’ nasal cavities that correlate positively with commensal microbiota abundance, while Trp depletion associates with compromised mucosal immunity through impaired neutrophil recruitment and pathogen clearance. Mechanistically, IL4I1-mediated Trp metabolism generates bioactive indole derivatives, particularly indole-3-aldehyde and indole-3-pyruvate, which function as endogenous AhR ligands. These metabolites exhibit immunomodulatory properties, with indole-3-aldehyde specifically enhancing CD8+ T cell immunosuppressive activity via AhR activation. This metabolic reprogramming appears to influence mucosal immunity through Th17/Treg balance modulation, suggesting a potential therapeutic target for CRS management. We hypothesize that Trp, indole-3-pyruvate, and indole-3-aldehyde metabolic cascade represents a critical pathway for IL4I1-AhR activation in CRS. These findings underscore the critical role of AhR signaling in mediating environmental pollutant-induced EMT and its implications for respiratory diseases. While this study offers valuable insights into the significant roles of AhR and IL4I1 in CRS, several limitations must be acknowledged. Firstly, our findings are predominantly based on in vitro cellular experiments and murine models. Future studies should focus on clinical validation to determine the exact functions of these molecules in human CRS pathogenesis. We are currently employing HPLC-MS/MS to measure Trp and its metabolites in nasal lavage samples from CRS patients, while simultaneously analyzing potential environmental interactions through correlation with satellite-monitored PM2.5 exposure levels. Then, the exact mechanisms by which IL4I1 regulates AhR expression through the modulation of Trp catabolism remain incompletely elucidated. Additional investigations are necessary to clarify the interactions between IL4I1 and AhR and to delineate their specific contributions to the pathogenesis of CRS.

## 5. Conclusions

In summary, our study underscores the central role of the AhR and its associated signaling pathways in the pathogenesis of CRS, particularly in mediating PM2.5-induced disruption of the nasal mucosal barrier and the promotion of EMT. We elucidated how PM2.5 stimulates human nasal mucosal epithelial, activate IL4I1, modulates AhR expression, and induces nasal mucosal damage and tissue remodeling, ultimately contributing to nasal polyp formation. Future research should focus on further unraveling the molecular mechanisms underlying the IL4I1-AhR signaling axis and developing targeted therapeutic strategies to modulate this pathway, thereby mitigating CRS progression and improving clinical outcomes.

## Data Availability

The raw data supporting the conclusions of this article will be made. available by the authors upon request.

## Supplementary Materials

The following supporting information can be downloaded at: https://www.mdpi.com/article/10.3390/toxics13060488/s1; Table S1: The list of primer sequences for target genes; Table S2: The key enzyme in tryptophan metabolism associated with AhR.

## Abbreviations

The following abbreviations are used in this manuscript:

CRS	chronic rhinosinusitis
CRSwNP	chronic rhinosinusitis with nasal polyps
CRSsNP	chronic rhinosinusitis without nasal polyps
EPOS	European Position Paper on Rhinosinusitis and Nasal Polyps
AhR	aryl hydrocarbon receptor
EMT	epithelial–mesenchymal transition
PAHs	Polycyclic Aromatic Hydrocarbons
KYN	kynurenine
IL4I1	Interleukin 4 Induced 1
LAAO	amino acid oxidase
BAP	Benzo[a]pyrene
NSAIDs	nonsteroidal anti-inflammatory drugs
IHC	immunohistochemistry
qRT-PCR	qualitative real-time polymerase chain reaction
WB	Western blot
HE	hematoxylin and eosin
HNEpCs	Human Nasal Mucosal Epithelial Cells
OD	Optical density
PPI	Protein–Protein Interaction
SD	standard deviation
ANOVA	one-way analysis of variance
LSD	least significant difference
DEHP	di(2-ethylhexyl) phthalate
